# The Link between *Plasmodium falciparum* Malaria and Endemic Burkitt’s Lymphoma—New Insight into a 50-Year-Old Enigma

**DOI:** 10.1371/journal.ppat.1005331

**Published:** 2016-01-21

**Authors:** David Thorley-Lawson, Kirk W. Deitsch, Karen A. Duca, Charles Torgbor

**Affiliations:** 1 Sackler School of Graduate Biomedical Sciences, Tufts University School of Medicine, Boston, Massachusetts, United States of America; 2 Department of Microbiology and Immunology, Weill Medical College of Cornell University, New York, New York, United States of America; 3 Department of Biochemistry and Biotechnology, Kwame Nkrumah University of Science and Technology (KNUST) and Kumasi Centre for Collaborative Research, Kumasi, Ghana; University of Wisconsin Medical School, UNITED STATES

## Introduction

The link between *Plasmodium falciparum* malaria and endemic Burkitt’s lymphoma (eBL) has been an enigma for more than 50 years, since it was first observed that the occurrence of the two coincided [1,2]. So convincing was the association that it led to the prediction that eBL was caused by an infectious agent that was spread by malarial mosquitoes. The subsequent search turned up the first human oncogenic virus, Epstein-Barr virus (EBV) [3,4]. EBV is found in nearly all cases of eBL [5] and has been shown to be a potent transforming virus for human B cells [4]. eBL is believed to arise from germinal center (GC) B cells [6] and is characterized by a typical translocation of the c-myc oncogene into one of the immunoglobulin loci [7,8]. The subsequent deregulation of c-myc expression would normally lead to rapid apoptosis of the cell, but the presence of EBV is thought to rescue these cells and allow them to survive [9]. The enigma remained, however, because EBV is not spread by mosquitoes. eBL therefore represents an intriguing and unusual situation in which interactions between a protozoan parasite (*P*. *falciparum*) and a mammalian virus (EBV) combine to cause cancer. While the mechanisms linking EBV to lymphoma development are becoming better understood, the link between *P*. *falciparum* malaria and eBL has remained completely unexplained until now. Finally, we have a breakthrough in this longstanding issue from two separate studies: one through investigation of human tissues and one in a mouse model.

## A Role for Activation-Induced Cytidine Deaminase (AID)

**Fig 1:**
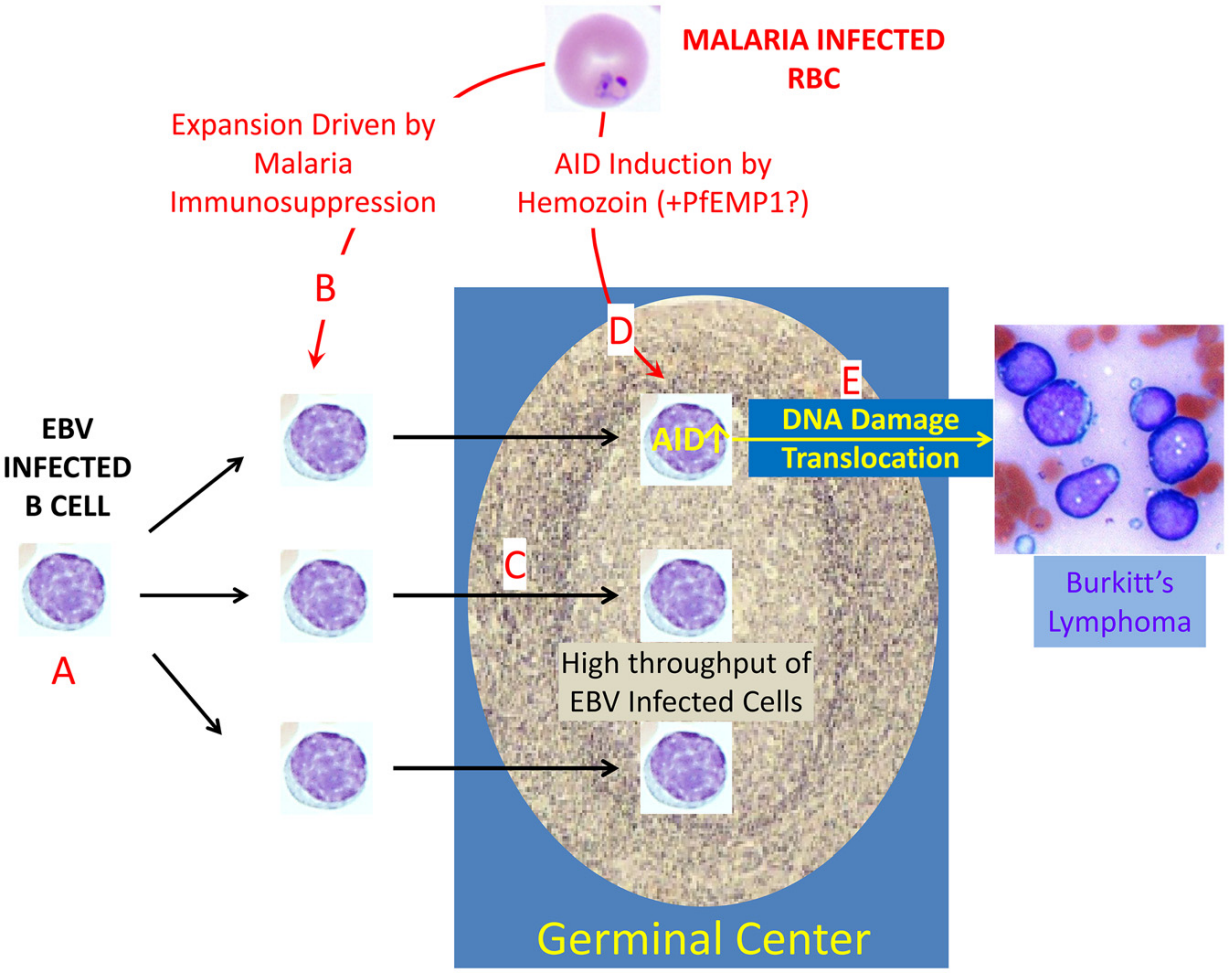
How *P*. *falciparum* increases the risk of endemic Burkitt’s lymphoma. Essentially all adults are persistently infected with EBV (A). As a consequence, newly infected B cells are continually being produced that transit the GC on their way to becoming latently infected memory B cells (the site of viral persistence) [14]. Malaria is immunosuppressive (B) [16,17], and Torgbor et al. have shown that this results in a highly elevated throughput of EBV-infected cells in the GC (C). Torgbor et al. also showed that *P*. *falciparum* induces deregulated expression of the DNA-mutating and -cutting enzyme AID in GC cells (D). Robbiani et al. subsequently showed in a mouse model that this deregulated expression led to DNA damage, translocations, and, ultimately, lymphoma (E). Thus, infection with *P*. *falciparum* has been shown to have two effects on the GC, where eBL originates. Together, these increase the risk that a GC cell will undergo a c-myc translocation and that this cell will also be EBV-infected and, therefore, able to tolerate the translocation, synergistically increasing the likelihood that eBL will arise.

The first breakthrough occurred with the demonstration that *P*. *falciparum* induces the DNA-mutating and double-strand-breaking enzyme activation-induced cytidine deaminase (AID) [10]. This is the enzyme that is normally responsible for the somatic hypermutation and class-switch recombination of immunoglobulin genes that occur in B cells when they enter the GC [11]. However, AID is also known to be somewhat promiscuous and occasionally mutate off targets, including oncogenes [12]. Usually, these mutations are repaired, but when AID expression is deregulated in mouse models, it becomes a risk factor for lymphoma development, including the c-myc translocation characteristic of eBL [13]. The study by Torgbor and colleagues utilized a unique sample of primary human tissues, specifically tonsils, obtained from individuals either chronically infected or uninfected with *P*. *falciparum*. This enabled them to isolate GC B cells directly from individuals at risk for developing eBL. They went on to show that individuals chronically infected with *P*. *falciparum* malaria had higher numbers of GC B cells that expressed elevated levels of AID and, furthermore, had an extremely high level of GC B cells latently infected with EBV [10]. To explain the mechanism behind the induction of AID in the tonsils of individuals with malaria, the authors observed that extracts from *P*. *falciparum*-infected red blood cells directly caused a strong activation of AID in tonsil B cells in vitro. They additionally showed that this was due, at least in part, to the action of hemozoin, the metabolic product of hemoglobin digestion by *P*. *falciparum* parasites. Together, these results allowed the investigators to conclude that *P*. *falciparum* infection increases two major risk factors for lymphoma development. The first is induction of AID in GC B cells, putting them at increased risk for a translocation; the second is a much higher frequency of EBV-infected cells in the GC, increasing the chances that the translocation will occur in a cell that will tolerate it (Fig 1). The increased combinatorial risk of these two events explains the increased prevalence of eBL in *P*. *falciparum*-endemic areas, but many questions remain. These include why eBL is specifically linked only with *P*. *falciparum* and not the other species that cause malaria in humans (discussed in detail below), the origins of sporadic BL (which is not linked with *P*. *falciparum* and is frequently EBV-negative), why eBL is predominantly a cancer of children, and why the tumor is located in very specific anatomical regions that tend to change with age (the jaw in children and the abdomen in adults). Nevertheless, these experiments represent an important breakthrough in our understanding of how *P*. *falciparum* and EBV interact to cause eBL. However, the limitations inherent in working with human populations and the lack of an experimental animal model of the disease has hindered a more detailed investigation.

## A Mouse Model for eBL

The study described by Torgbor et al. predicted that *P*. *falciparum* infection would lead directly to AID-dependent DNA damage and translocations in the GC, ultimately producing lymphoma—a prediction impossible to test in an actual human infection. This prediction has now been confirmed by employing the *P*. *chabaudi* mouse model of malaria [14]. In this study, Robbiani et al. showed that mice repeatedly infected with this species of malaria parasite develop prolonged expansion of GCs in which the B cells undergo rapid expansion and express AID—exactly what was seen in the human infection with *P*. *falciparum*. These authors went on to show that this resulted in the accumulation of widespread DNA damage in the GC cells, including translocations, which was associated with subsequent lymphoma development, but only when expressed on a p53-null background. One might speculate that the p53 knockout essentially complemented the lack of EBV infection in the mice and in the GC B cells (but see caveat below). What was lacking in this study was evidence that the rodent parasite *P*. *chabaudi* was in any way an accurate model for *P*. *falciparum* and, specifically, that the rapid expansion of GC B cells and induction of AID expression are actually seen in the human disease. This was conveniently provided by the Torgbor et al. study.

### The Specificity of eBL for Infection by *P*. *falciparum*—Limitations of the Mouse Model

While the mouse studies provide a potential animal model for eBL, there remain serious concerns, most notably with respect to specificity. Human malaria is caused by five different species of Plasmodium; however, eBL is specifically associated with *P*. *falciparum* and is primarily observed in regions of the world endemic for this species of parasite, namely sub-Saharan Africa and Papua New Guinea. While *P*. *falciparum* generally causes a more severe form of malaria, all species can cause chronic infections, and, thus, the reason for its specific association with eBL remains unanswered. Given this specificity, it is reasonable to question if *P*. *chabaudi* is an accurate model of *P*. *falciparum* infection. The Torgbor et al. study implicated hemozoin as playing a role in inducing AID expression in GC B cells through Toll-like receptor (TLR) signaling, but this mechanism was not tested in the mouse study. Furthermore, hemozoin alone was not as effective as complete extracts obtained from *P*. *falciparum*-infected red blood cells, suggesting other components also play a role.

Recent studies have implicated the *P*. *falciparum*-specific protein PfEMP1 in polyclonal B cell activation and increased survival, leading the authors to propose a possible link to eBL [15]. PfEMP1 is an antigenic protein expressed on the surface of infected red blood cells that is unique to *P*. *falciparum*; thus, if PfEMP1 plays a key role in eBL, it could explain the specificity of the disease to *P*. *falciparum*. Like all Plasmodium strains, the rodent parasites, including *P*. *chabaudi*, express antigenic proteins on the infected red blood cell surface, but they are unrelated to PfEMP1. If it can be shown that PfEMP1 does indeed play an important role in the development of eBL, it would raise significant questions about the specificity of the mouse model.

A second concern with the mouse model is that it was necessary to perform the infection on a p53-/- background. The p53-/- strain alone produces lymphoma, and infection with *P*. *chabaudi* did not increase the incidence of lymphoma but only altered the tumors to a more mature phenotype. This is in contrast with *P*. *falciparum*, for which compelling epidemiological evidence has demonstrated that it dramatically increases the occurrence of eBL. Taken together, these observations suggest the mouse model may mimic only part of the human disease and lacks the specificity associated with *P*. *falciparum*.

## Perspectives

In summary, therefore, two recent, very complementary papers finally provide the missing link between infection with *P*. *falciparum* malaria and the development of eBL. The first paper [10] demonstrated that the human pathogen actually induces AID expression in the disease setting, but it could only speculate on the link between this induction of AID and the DNA damage leading to lymphoma. The subsequent paper [14] extended this result to show, in a mouse model, that AID induced by malaria was indeed a risk factor for DNA damage and lymphoma, but it could only speculate that this mechanism actually occurred in and was directly related to the human disease. Taken together, these two independent studies demonstrate the power of combining the direct study of human disease to validate animal models that can then be used to develop a more detailed understanding. The first significant insights into this 50-year-old enigma have finally come to light—*P*. *falciparum* malaria is a risk factor for eBL because it drives a high throughput of EBV-infected cells through the GC, where it also deregulates AID, leading to DNA damage, c-myc translocations, and lymphoma (Fig 1).

## References

[1] Burkitt D (1962) A children's cancer dependent on climatic factors. Nature 194: 232–234. 13874900 10.1038/194232a0

[2] Morrow RH Jr. (1985) Epidemiological evidence for the role of falciparum malaria in the pathogenesis of Burkitt's lymphoma. IARC Sci Publ: 177–186. 3905588

[3] Epstein MA, Achong BG, Barr YM, Zajac B, Henle G, et al (1966) Morphological and virological investigations on cultured Burkitt tumor lymphoblasts (strain Raji). J Natl Cancer Inst 37: 547–559. 4288580

[4] Henle W, Diehl V, Kohn G, Zur Hausen H, Henle G (1967) Herpes-type virus and chromosome marker in normal leukocytes after growth with irradiated Burkitt cells. Science 157: 1064–1065. 6036237 10.1126/science.157.3792.1064

[5] Magrath I (1990) The pathogenesis of Burkitt's lymphoma. Adv Cancer Res 55: 133–270. 2166998 10.1016/s0065-230x(08)60470-4

[6] Klein U, Dalla-Favera R (2008) Germinal centres: role in B-cell physiology and malignancy. Nat Rev Immunol 8: 22–33. 18097447 10.1038/nri2217

[7] Hecht JL, Aster JC (2000) Molecular biology of Burkitt's lymphoma. J Clin Oncol 18: 3707–3721. 11054444 10.1200/JCO.2000.18.21.3707

[8] Klein G (1983) Specific chromosomal translocations and the genesis of B-cell-derived tumors in mice and men. Cell 32: 311–315. 6402307 10.1016/0092-8674(83)90449-x

[9] Thorley-Lawson DA, Allday MJ (2008) The curious case of the tumour virus: 50 years of Burkitt's lymphoma. Nat Rev Microbiol 6: 913–924. 10.1038/nrmicro2015 19008891

[10] Torgbor C, Awuah P, Deitsch K, Kalantari P, Duca KA, et al (2014) A multifactorial role for P. falciparum malaria in endemic Burkitt's lymphoma pathogenesis. PLoS Pathog 10: e1004170 10.1371/journal.ppat.1004170 24874410 PMC4038605

[11] Muramatsu M, Kinoshita K, Fagarasan S, Yamada S, Shinkai Y, et al (2000) Class switch recombination and hypermutation require activation-induced cytidine deaminase (AID), a potential RNA editing enzyme. Cell 102: 553–563. 11007474 10.1016/s0092-8674(00)00078-7

[12] Liu M, Duke JL, Richter DJ, Vinuesa CG, Goodnow CC, et al (2008) Two levels of protection for the B cell genome during somatic hypermutation. Nature 451: 841–845. 10.1038/nature06547 18273020

[13] Ramiro AR, Jankovic M, Eisenreich T, Difilippantonio S, Chen-Kiang S, et al (2004) AID is required for c-myc/IgH chromosome translocations in vivo. Cell 118: 431–438. 15315756 10.1016/j.cell.2004.08.006

[14] Robbiani DF, Deroubaix S, Feldhahn N, Oliveira TY, Callen E, et al (2015) Plasmodium Infection Promotes Genomic Instability and AID-Dependent B Cell Lymphoma. Cell 162: 727–737. 10.1016/j.cell.2015.07.019 26276629 PMC4538708

[15] Simone O, Bejarano MT, Pierce SK, Antonaci S, Wahlgren M, et al (2011) TLRs innate immunereceptors and Plasmodium falciparum erythrocyte membrane protein 1 (PfEMP1) CIDR1alpha-driven human polyclonal B-cell activation. Acta Trop 119: 144–150. 10.1016/j.actatropica.2011.05.005 21620790 PMC3406598

[16] Moormann AM, Chelimo K, Sumba PO, Tisch DJ, Rochford R, et al (2007) Exposure to holoendemic malaria results in suppression of Epstein-Barr virus-specific T cell immunosurveillance in Kenyan children. J Infect Dis 195: 799–808. 17299709 10.1086/511984

[17] Moormann AM, Chelimo K, Sumba OP, Lutzke ML, Ploutz-Snyder R, et al (2005) Exposure to holoendemic malaria results in elevated Epstein-Barr virus loads in children. J Infect Dis 191: 1233–1238. 15776368 10.1086/428910

